# AGE-RELATED KYNURENINE PATHWAY METABOLITES DRIVE SENESCENT-LIKE CHANGES IN NEURONS: POTENTIAL NOVEL AD MECHANISM

**DOI:** 10.1093/geroni/igad104.3712

**Published:** 2023-12-21

**Authors:** Dmitry Kondrikov, Galina Kondrikova, Steven Dixon, Carsten Krieg, Gavin Wang, William Hill

**Affiliations:** Medical University of South Carolina, Charleston, South Carolina, United States; Medical University of South Carolina, Charleston, South Carolina, United States; Medical University of South Carolina, Charleston, South Carolina, United States; Medical University of South Carolina, Charleston, South Carolina, United States; Medical University of South Carolina, Charleston, South Carolina, United States; Medical University of South Carolina, Charleston, South Carolina, United States

## Abstract

Kynurenine pathway (KP) metabolites are emerging as important factors of aging-related pathologies. Kynurenine (KYN) and its metabolites increase with age and are known to affect multiple organ systems including CNS, musculoskeletal, and vascular systems. Our group has previously shown that KYN and sub-KYN metabolites below the enzyme Kynurenine monooxygenase (KMO) can act to inhibit osteogenesis of bone marrow MSCs via senescence induction. However, inhibition of KMO, blocks KYN induced senescence and restores osteogenic potentials of MSCs. We are now expanding this work to see if KYN metabolites act on cells of the aging CNS in a similar fashion potentially helping to drive cognitive decline and Alzheimer’s disease (AD) progression. We report for the first time that treatment of neuroblastoma-like SH-SY5Y cells with KYN, and sub-KMO metabolites 3-hydroxykynurenine (3HK), or quinolinic acid (QA) significantly increased senescence-associate beta-galactosidase activity, and expression of senescence associated secretory phenotype (SASP) markers, p21, PAI-1, TIMP2 as well as Histone 3 K9methylation. Treatment of SH-SY5Y cells with KMO inhibitors prevented elevated expression of SASP proteins and histone methylation. This makes KMO an attractive target to inhibit kynurenine pathway effects. Taken together, these data suggest that KYN and its metabolites may contribute to pathogenesis of AD via inducing senescence-like changes and SASP-mediated neuroinflammation in neuronal cells. Inhibition of KMO may allow rescue of brain microenvironment, limit cognitive decline and AD progression in older persons. Blocking KYN downstream metabolite formation, by inhibiting KMO, presents a novel therapeutic target for a potential novel AD mechanism.

